# Author Correction: Steering surface reconstruction of copper with electrolyte additives for CO_2_ electroreduction

**DOI:** 10.1038/s41467-022-31696-4

**Published:** 2022-07-13

**Authors:** Zishan Han, Daliang Han, Zhe Chen, Jiachen Gao, Guangyi Jiang, Xinyu Wang, Shuaishuai Lyu, Yong Guo, Chuannan Geng, Lichang Yin, Zhe Weng, Quan-Hong Yang

**Affiliations:** 1 Nanoyang Group, State Key Laboratory of Chemical Engineering, School of Chemical Engineering and Technology, Tianjin University, Tianjin, 300072 China; 2 Haihe Laboratory of Sustainable Chemical Transformations, Tianjin, 300192 China; 3 Shenyang National Laboratory for Materials Science, Institute of Metal Research, Chinese Academy of Sciences, 72 Wenhua Road, Shenyang, 110016 China; 4 Department of Physics and Electronic Information, Huaibei Normal University, Anhui, Huaibei, 235000 China; 5 Joint School of National University of Singapore and Tianjin University, International Campus of Tianjin University, Binhai New City, Fuzhou, 350207 China

**Keywords:** Electrocatalysis, Electrocatalysis, Electrocatalysis

Correction to: *Nature Communications* 10.1038/s41467-022-30819-1, published online 07 June 2022.

The original version of this Article contained an error in Fig. 5, in which the reference number used in the Fig. 5c did not match with that used in the legend. This has been corrected in both the PDF and HTML versions of the Article.

