# Supplementary material for: Steering surface reconstruction of copper with electrolyte additives for CO2 electroreduction
Source: Nat Commun. 2022 Jun 7;13:3158. doi: 10.1038/s41467-022-30819-1 (PMC9174297; doi:10.1038/s41467-022-30819-1)
Supplement: Supplementary file 1 — Supplementary Information [file 41467_2022_30819_MOESM1_ESM.pdf]

Supplementary Information

Steering surface reconstruction of copper with electrolyte additives for CO_2 electroreduction

Zishan Han[1,2,6], Daliang Han[1,2,6], Zhe Chen[3,6], Jiachen Gao[1,2], Guangyi Jiang[1,2], Xinyu Wang[1,2], Shuaishuai Lyu[1,2], Yong Guo[1,2], Chuannan Geng[1,2], Lichang Yin[3,4]*, Zhe Weng[1,2]* and Quan-Hong Yang[1,2,5]*

[1]Nanoyang Group, State Key Laboratory of Chemical Engineering, School of Chemical Engineering and Technology, Tianjin University, Tianjin 300072, China

[2]Haihe Laboratory of Sustainable Chemical Transformations, Tianjin 300192, China

[3]Shenyang National Laboratory for Materials Science, Institute of Metal Research, Chinese Academy of Sciences, 72 Wenhua Road, Shenyang 110016, China

[4]Department of Physics and Electronic Information, Huaibei Normal University, Anhui, Huaibei, 235000, China

[5]Joint School of National University of Singapore and Tianjin University, International Campus of Tianjin University, Binhai New City, Fuzhou 350207, China

[6]These authors contributed equally.

E-mail: lcyin@imr.ac.cn; zweng@tju.edu.cn; qhyangcn@tju.edu.cn

Supplementary Figures

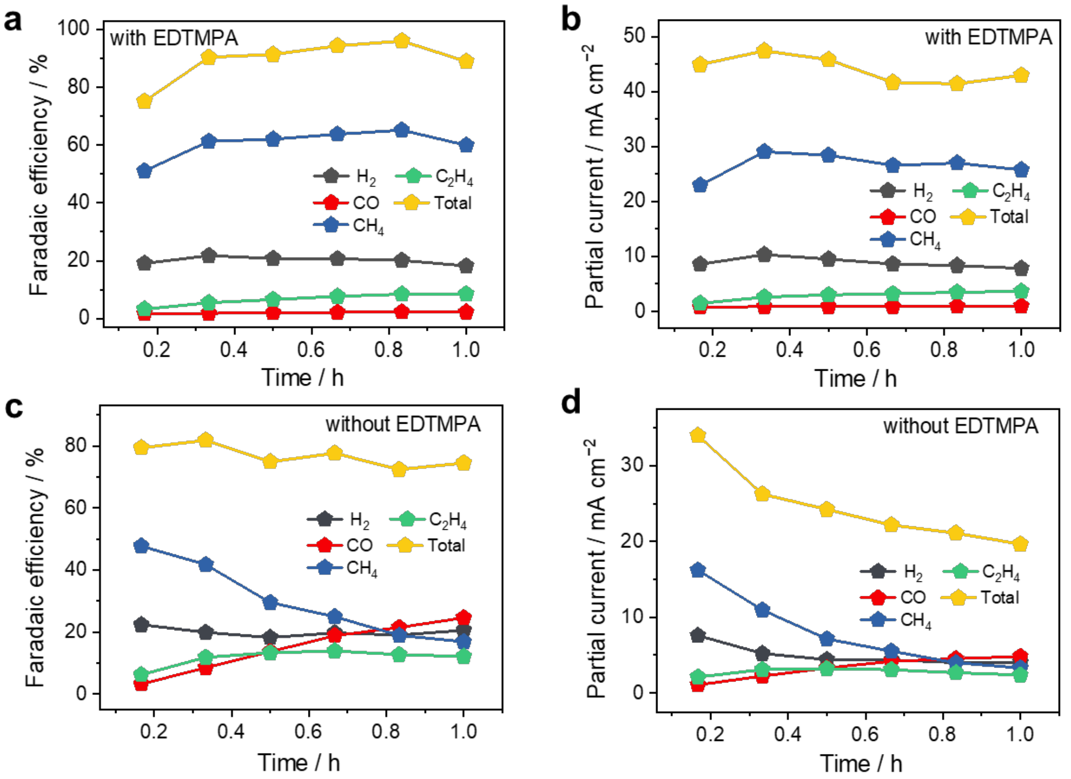

Supplementary Fig. 1 | Stability of electrocatalytic CO_2 reduction in an H-cell. a–d, CO_2 electroreduction at −1.0 V versus RHE for 1 h on a poly-Cu electrode in 0.5 M $KHCO_3$ with (**a**, **b**) and without 8 ppm EDTMPA (**c**, **d**).

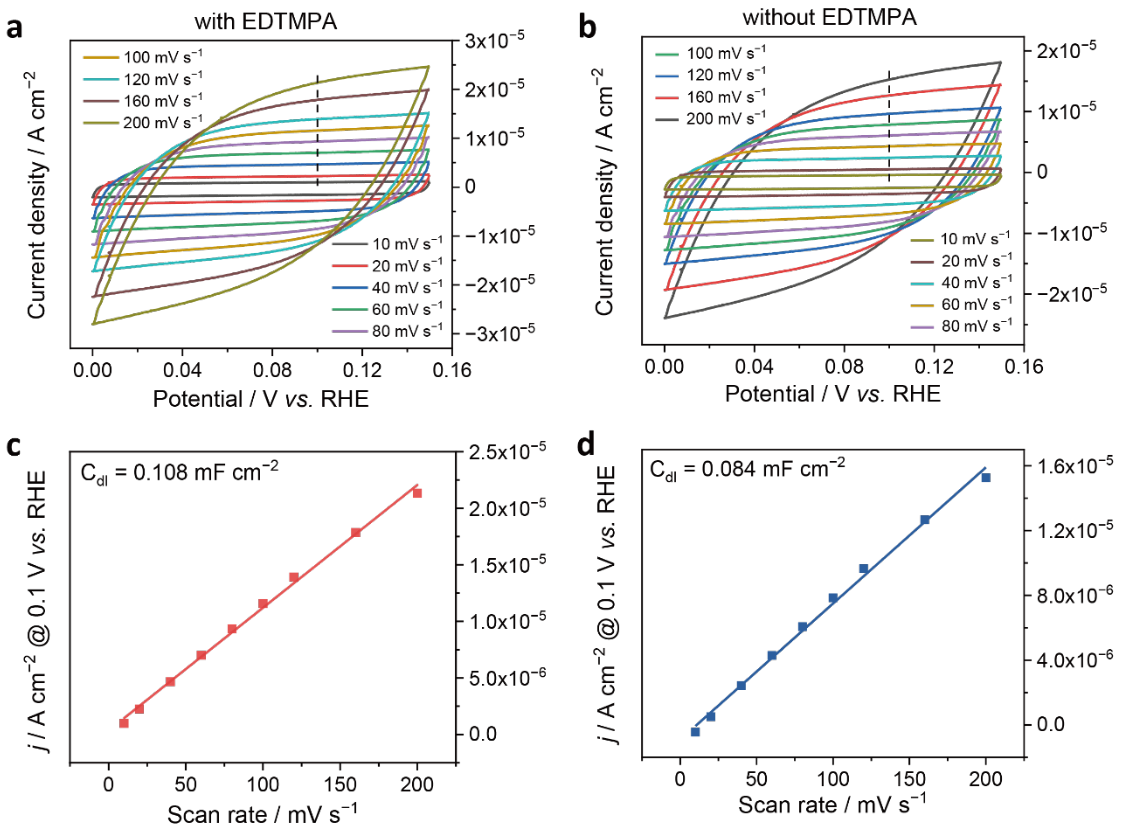

Supplementary Fig. 2 | Characterization of surface roughness factors. a, b, Scan-rate dependence of cyclic voltammetric stripping. CV curves over poly-Cu electrodes at different scan rates ranging from 10 to 200 mV s^{-1} with (**a**) and without (**b**) EDTMPA were performed in a 0.5 M $KHCO_3$ solution to calculate the C_{dl} of each catalyst. **c, d**, Current density of CV experiments at a potential of 0.1 V versus RHE as a function of scan rate. The slopes of the lines in (**c**) and (**d**) indicate the double-layer capacitances for the poly-Cu electrode with and without EDTMPA, respectively. The surface roughness factors (R_f) of the poly-Cu electrode with and without EDTMPA were estimated to be 1.61 and 1.25, respectively.

The R_f of poly-Cu electrodes tested with and without the addition of EDTMPA were determined by measuring the double layer capacitance (C_{dl}). The results show that the Cu surface of the case with EDTMPA is about 1.28 times rougher than that without EDTMPA. The slightly rougher Cu surface of the former is due to the EDTMPA-induced poly-Cu surface reconstruction with uniformly distributed and well-defined small particles.

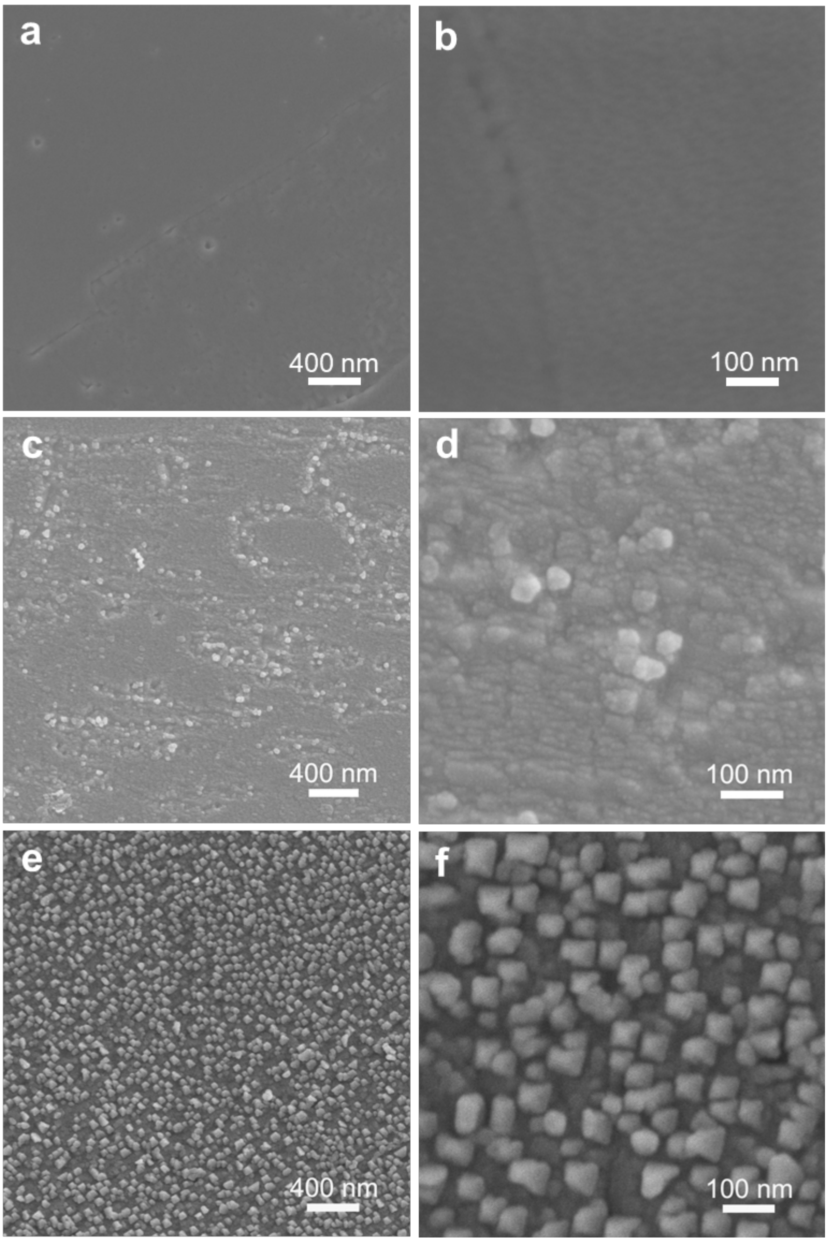

Supplementary Fig. 3 | SEM images of the poly-Cu electrodes before and after electrocatalysis. **a–f**, SEM images of a pristine poly-Cu electrode (**a**, **b**) and those after electrocatalysis in electrolytes without (**c**, **d**) and with EDTMPA (**e**, **f**).

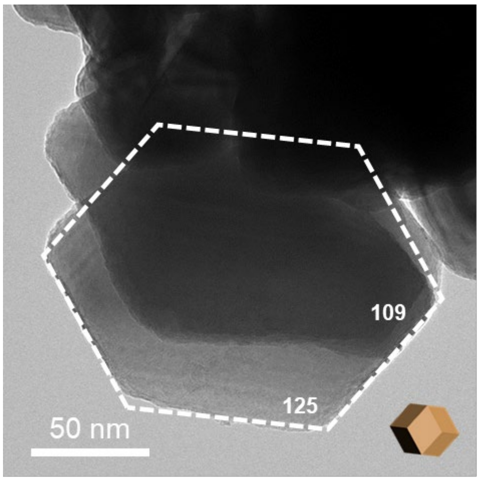

Supplementary Fig. 4 | TEM image of an electrodeposited Cu TEM grid after electrocatalysis in the electrolyte with EDTMPA.

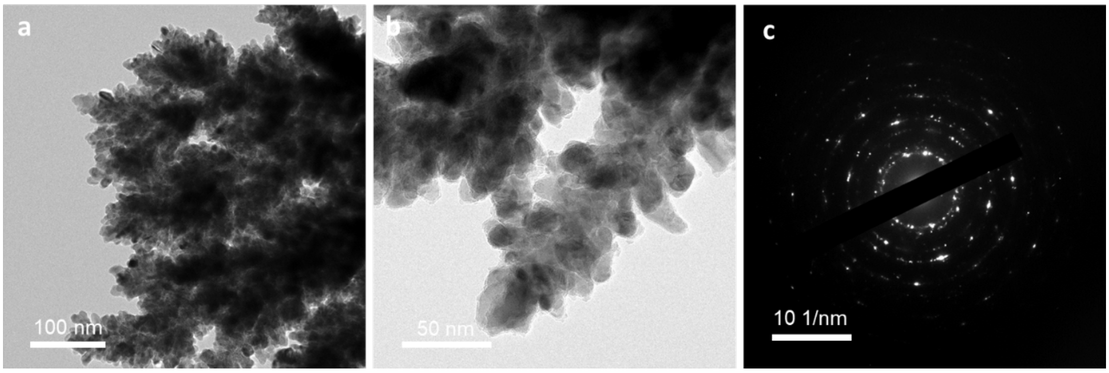

Supplementary Fig. 5 | TEM characterization of the as-electrodeposited Cu TEM grid. a, b, TEM images. **c,** The corresponding SAED pattern of **b**.

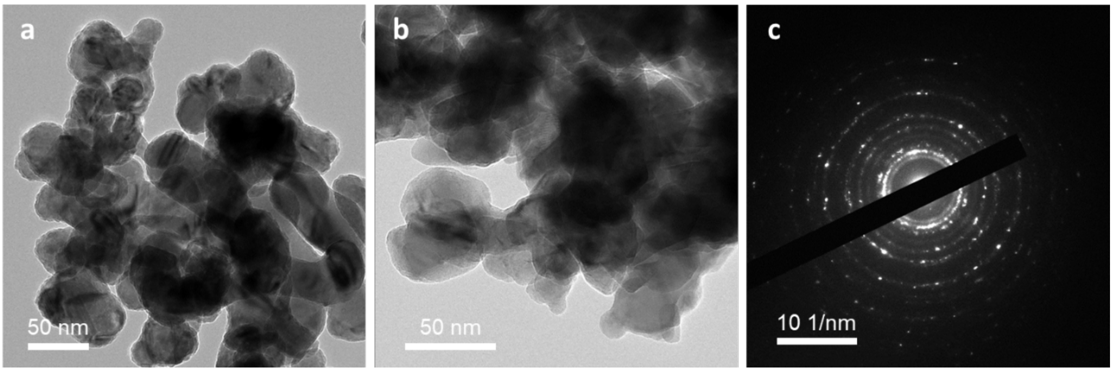

Supplementary Fig. 6 | TEM characterization of the electrodeposited Cu TEM grid after electrocatalysis in the electrolyte without EDTMPA. a, b, TEM images. **c,** The corresponding SAED pattern of **b**.

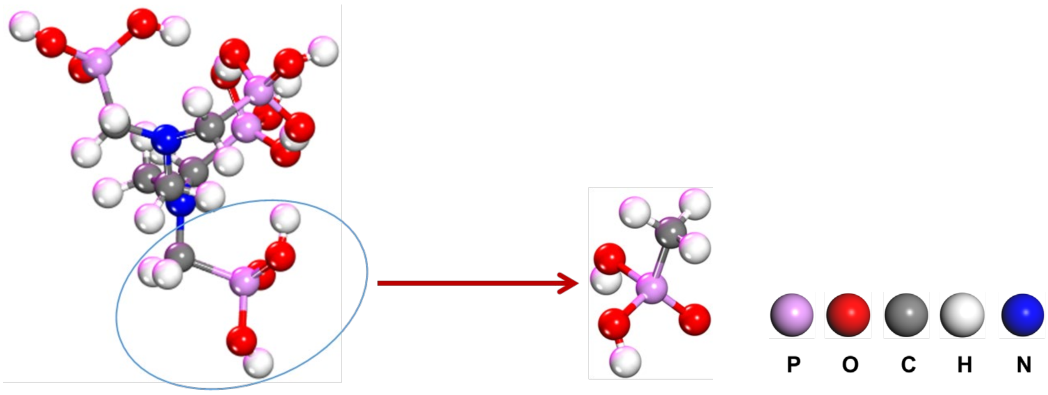

Supplementary Fig. 7 | Structure of the EDTMPA molecule. Atomic structures of the complete EDTMPA compound and the partial EDTMPA compound (MPA) used in the DFT calculations. The P, N, C, O and H atoms are denoted by purple, blue, grey, red and white balls, respectively.

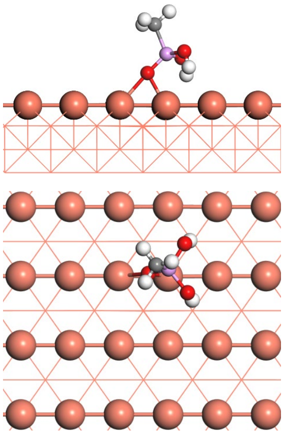

Supplementary Fig. 8 | Simulated model of MPA on Cu(110). The atomic structure of a *MPA molecule adsorbed on a Cu(110) surface, in which one O atom of the *MPA molecule bonds with two surface Cu atoms, and the bond lengths of two Cu−O bonds are calculated to be 2.10 and 2.36 Å, respectively.

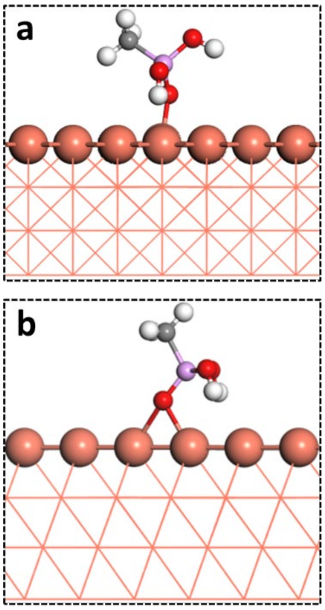

Supplementary Fig. 9 | Simulated models of MPA on Cu(100) and Cu(111). a, b, The atomic structures of MPA adsorbed on a Cu(100) (**a**) and a Cu(111) (**b**).

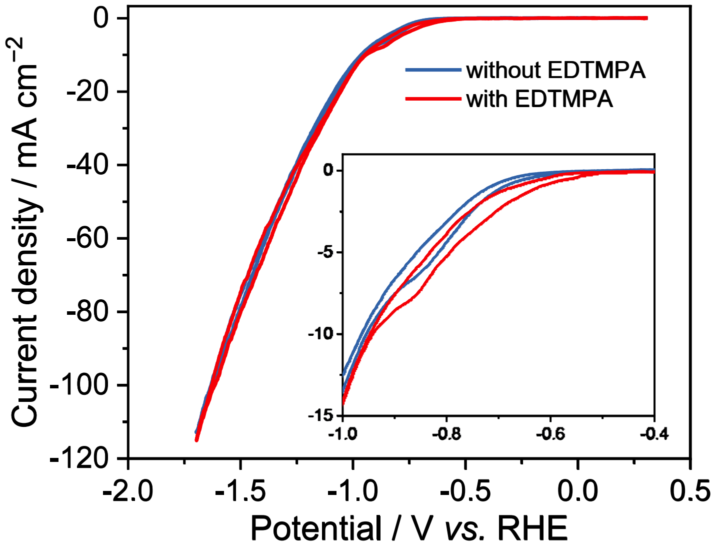

Supplementary Fig. 10 | The stability of EDTMPA molecules under bias. Comparison of CV curves between 0.3 V and −1.7 V versus RHE at a scan rate of 50 mV s^{-1} on poly-Cu electrodes in the Ar-saturated 0.5 M $KHCO_3$ electrolyte with and without 100 ppm EDTMPA.

As shown in the inset, both EDTMPA-added and EDTMPA-free cases exhibit the similar reduction wave, indicating it is unrelated to the adsorption/desorption or decomposition of EDTMPA. We speculate that the reduction wave indicates the change of dominant proton donors from HCO_3^- to H_2O[1], as suggested by the current plateaued where HCO_3^- reduction becomes limited by mass transport.

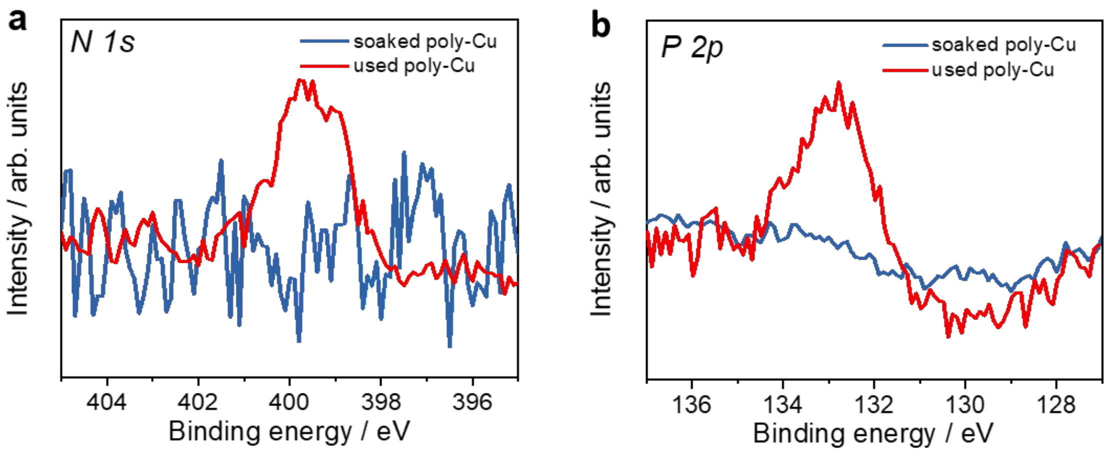

Supplementary Fig. 11 | Detection of EDTMPA on the Cu surface. a, b, N 1s (**a**) and P 2p (**b**) XPS spectra of the soaked poly-Cu electrode at open circuit potential and the used poly-Cu electrode after electrolysis at −1 V versus RHE in the presence of 8 ppm EDTMPA.

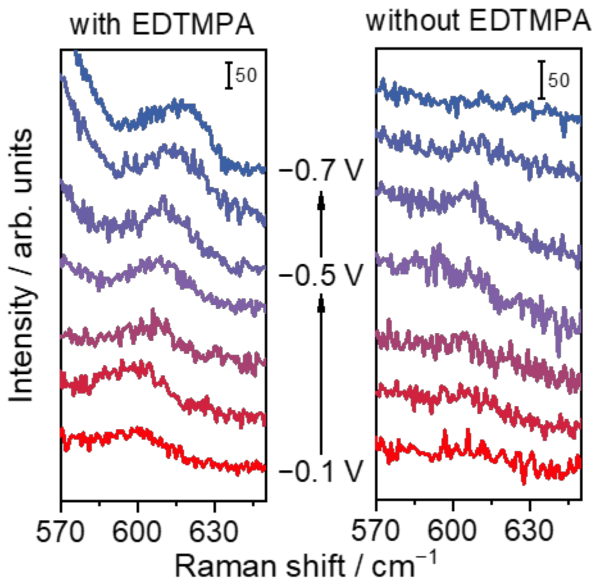

Supplementary Fig. 12 | *In situ* Raman spectra demonstrating the adsorption of EDTMPA on poly-Cu. *In-situ* Raman spectra of poly-Cu at potentials from −0.1 to −0.7 V versus RHE in a CO_2-saturated 0.5 M $KHCO_3$ solution with and without EDTMPA. The Raman shifts at 600−620 cm^{-1} correspond to the Cu−O stretching mode.

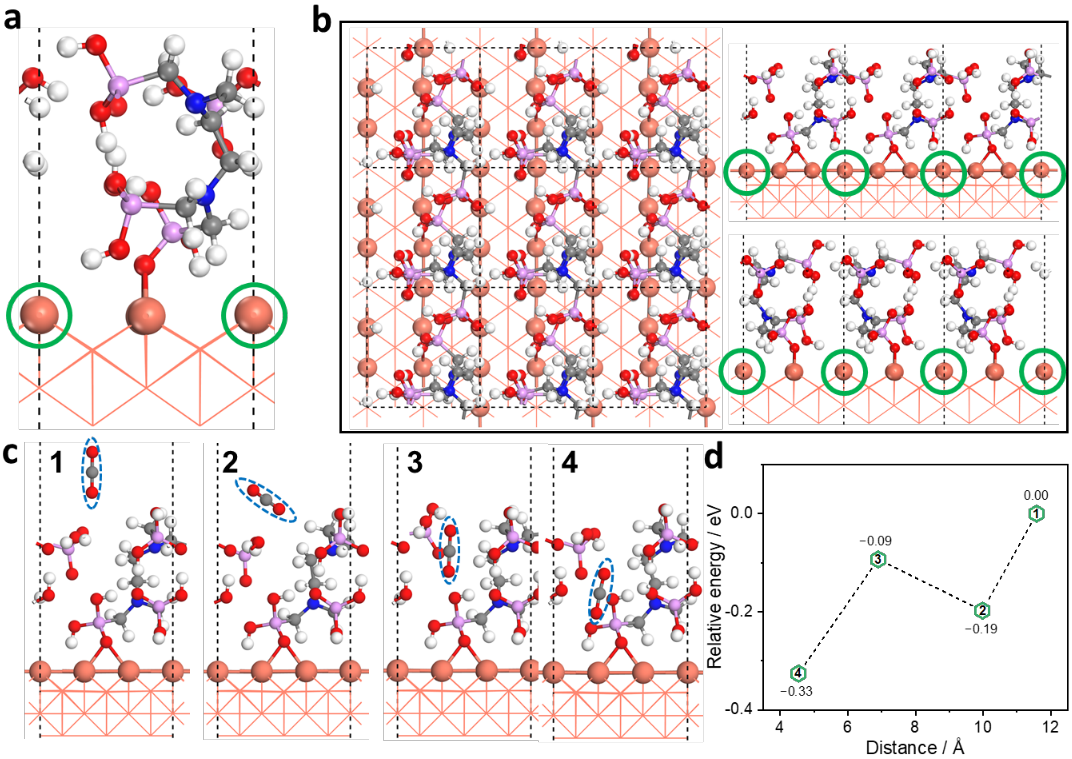

Supplementary Fig. 13 | Illustration of the coverage of EDTMPA molecules. a, Atomic structure of a single EDTMPA molecule adsorbed on the Cu(110) surface with a 2×3 supercell. **b**, Periodic display of **a** with different views. Note that the unoccupied Cu sites are marked with green circles. **c**, Atomic structure of a CO_2 molecule with different locations (configuration 1−4) from above EDTMPA to its interior. Note that the CO_2 molecules are highlighted with blue dashed circles for identification. **d**, Calculated relative energy with regard to the distance (configuration 1−4). The calculated total energy of configuration 1 was used as a reference (set to be zero), and the distance was defined as the distance between the carbon atoms of the CO_2 molecule and the Cu(110) surface.

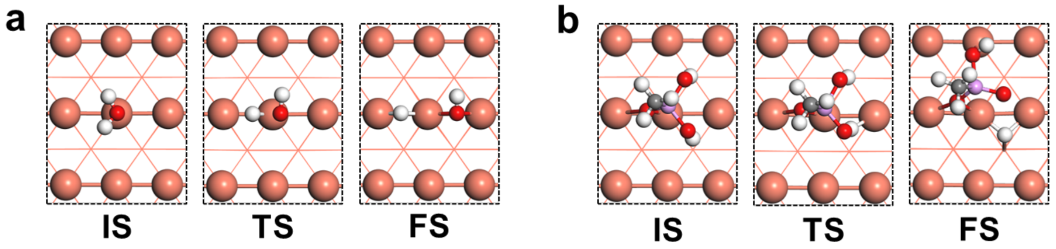

Supplementary Fig. 14 | Illustration of two different *H transfer paths from water and MPA molecules to the Cu(110) surface. a, b, Atomic structures of the initial state (IS), transition state (TS), and final state (FS) of the kinetic energy diagram of an H atom transferred from water (**a**) and MPA (**b**) to a clean Cu(110) surface.

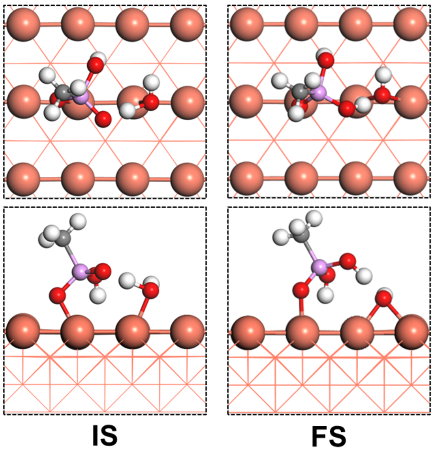

Supplementary Fig. 15 | Illustration of MPA capturing *H from H_2O. The atomic structures of an H atom transferred from an adsorbed H_2O (*H_2O) to MPA that loses one H (*MPA−H). Note that this transfer process is barrier-free, so only the atomic structures of the initial state (IS) and final state (FS) are given.

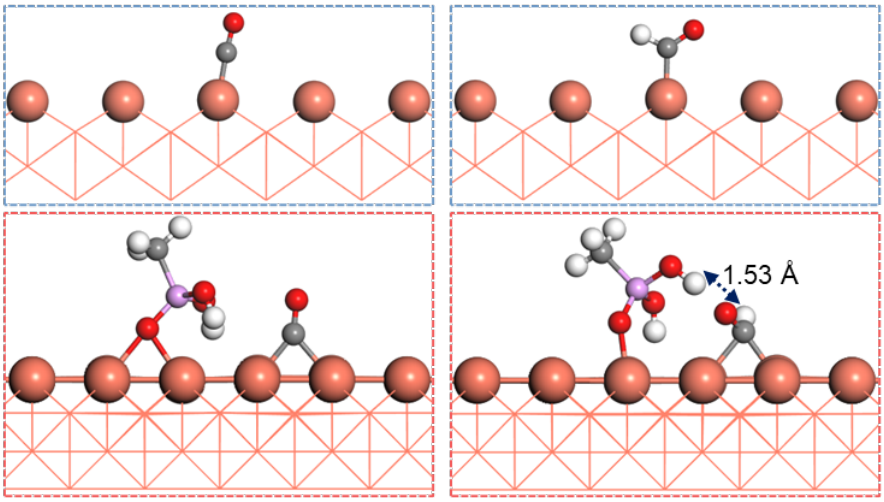

Supplementary Fig. 16 | Illustration of the formation of hydrogen bond between MPA and *CHO. The corresponding atomic structures of *CO and *CHO intermediate species in a Cu(110) surface without and with MPA molecule. The distance (1.53 Å) within the range of hydrogen bond (shorter than 3.0 Å)[2] is also marked.

Since the binding energies of the two key intermediates (*CO and *CHO) are proportional with a slope of 0.88[3], the stronger adsorption of *CHO would be accompanied by tighter binding of *CO to a similar extent, imposing restrictions on the independent optimization of the two bound adsorbates. Thus, the binding energies of the two species should be decoupled. In other words, the scaling relation between them should be broken to attain effective CO_2 electrocatalytic reduction to CH_4.

Based on previous calculations[4, 5], the key step of CO_2 electroreduction to CH_4 is the *CO species hydrogenation to the *CHO species. Our results show that the free energy change for this process is 0.79 eV on a clean Cu(110) surface, which is well consistent with a previous report[4]. After introducing EDTMPA onto the Cu(110) surface, *CHO species are more stably adsorbed on the surface through hydrogen bonding with the EDTMPA. Accordingly, the free energy change of *CO to *CHO is reduced from 0.79 to 0.41 eV, which means that EDTMPA can increase the electroreduction of CO_2 to CH_4 and break the linear relationship by stabilizing the key *CHO species through hydrogen bonds.

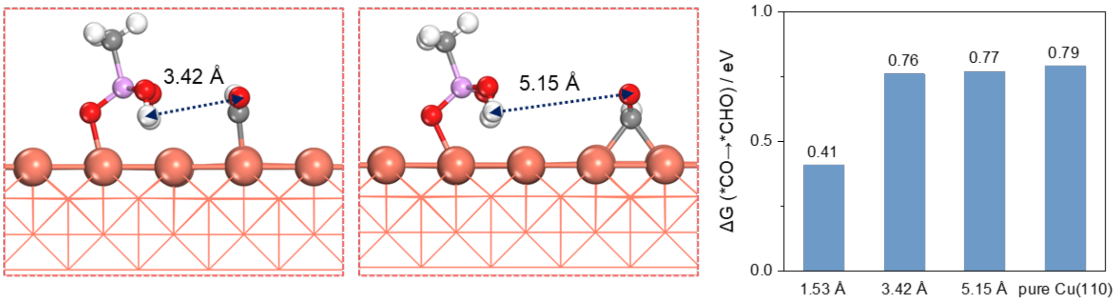

Supplementary Fig. 17 | Illustration of the range of hydrogen bond between MPA and *CHO. The corresponding atomic structures of *CHO intermediate species on the Cu(110) surface with MPA molecule with different distances between MPA and *CHO intermediate. And the energy change for *CO hydrogenation to *CHO, i.e., ΔE(*CO→*CHO) with different distances is provided for comparison.

A weak adsorption of *CHO intermediate can be observed when it is far away from the EDTMPA molecule. Correspondingly, the calculated energy change from *CO to *CHO is basically consistent with that on pure Cu(110) surface without EDTMPA molecule, suggesting no hydrogen bond is formed. Therefore, the above comparison indicates that the hydrogen bond is responsible for stabilizing the *CHO intermediate.

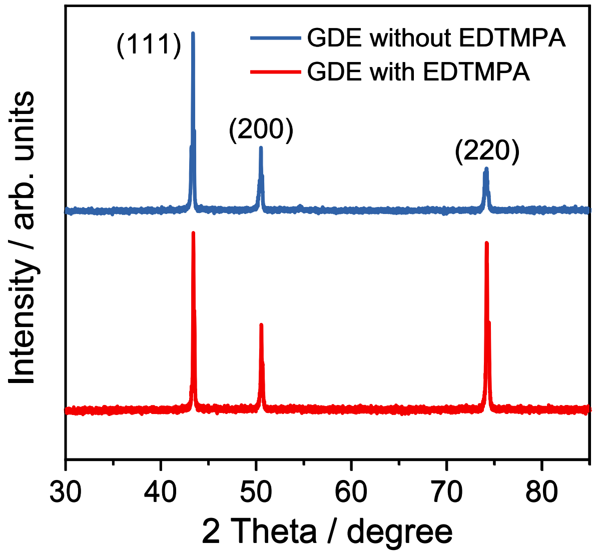

Supplementary Fig. 18 | GIXRD patterns of GDEs after electrocatalysis in alkaline electrolytes with and without EDTMPA.

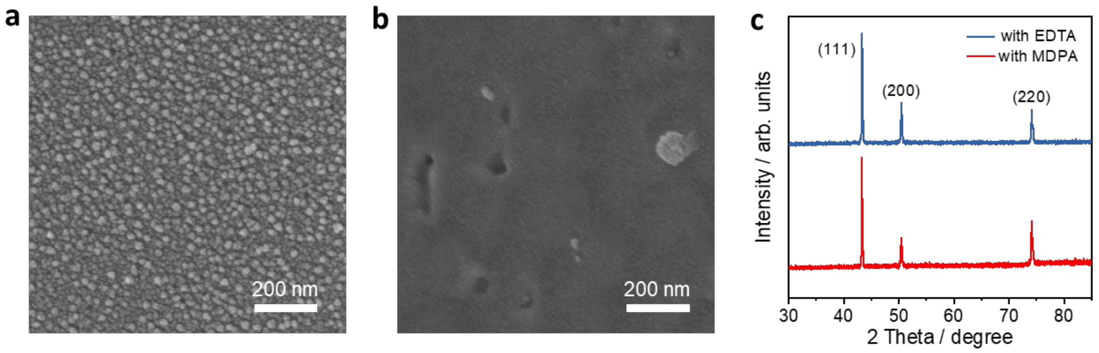

Supplementary Fig. 19 | Characterization of the poly-Cu electrodes with different additives. a, **b**, SEM images of poly-Cu electrodes after electrocatalysis in the electrolyte with MDPA (**a**) and EDTA (**b**). **c**, XRD patterns of poly-Cu electrodes after electrocatalysis in the electrolytes with MDPA and EDTA.

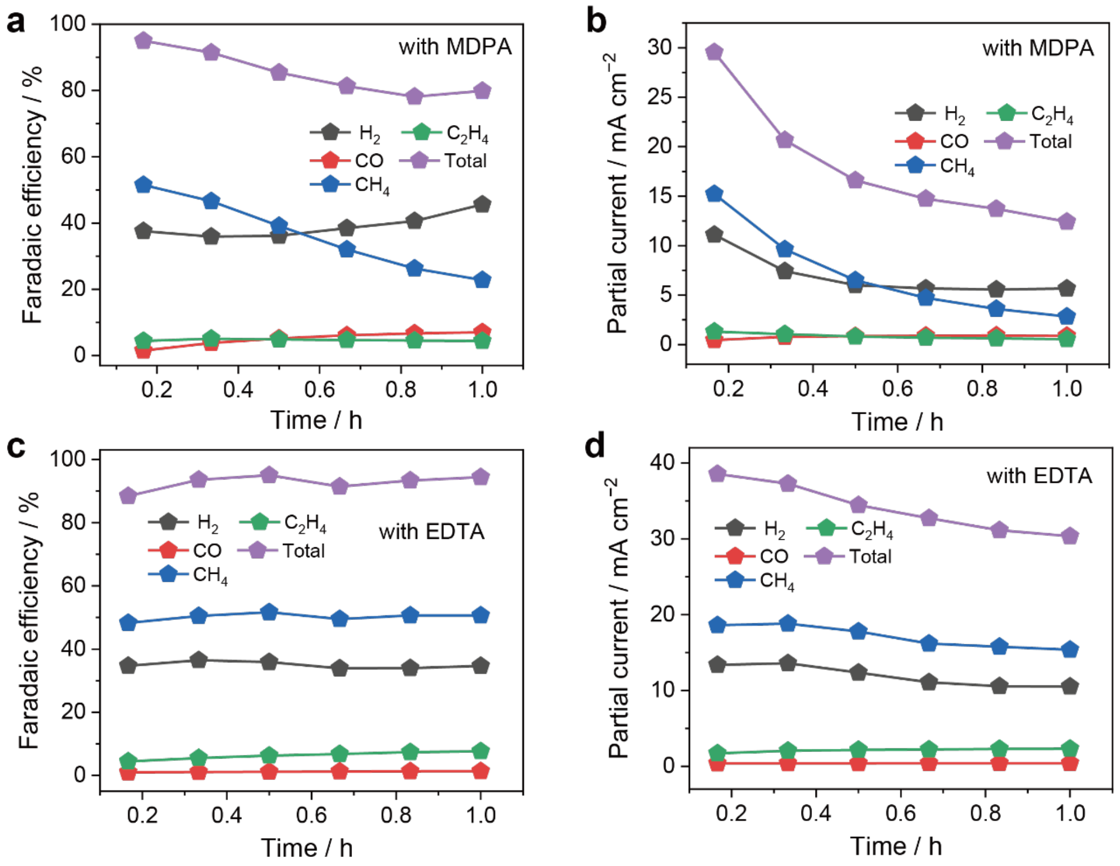

Supplementary Fig. 20 | CO_2 electroreduction performance with different additives. a, **b**, FEs (**a**) and partial current densities (**b**) of various products at −1.0 V versus RHE in the electrolytes with 8 ppm MDPA. **c**, **d**, FEs (**c**) and partial current densities (**d**) of various products at −1.0 V versus RHE in the electrolytes with 8 ppm EDTA.

Supplementary Table 1. The calculated adsorption energy of intact EDTMPA molecule on Cu(110) surface under different electric force fields.

Electric force field (V/Å)	−0.30	−0.20	−0.10	0.00
Adsorption energy (eV)	−2.54	−2.38	−2.25	−2.12

Clearly, the external electric field will increase the adsorption of EDTMPA molecules on Cu(110) surface. Note that assuming the width of the double layer is ~3 Å, the typical average field is ~0.3 V/Å at a potential of −1 V[6]. Thus, our DFT simulations cover the potentials in the experimental conditions.

Supplementary Table 2. The calculated total energies of *CHO and *COH intermediates on Cu(110) surface and the energy difference, without and with EDTMPA molecule (the unit is eV).

	pure (110) surface	with EDTMPA
*CHO	-357.670	-415.143
*COH	-356.956	-414.116
Energy difference	-0.714	-1.027

Although *CHO is widely known as the intermediate of *CO hydrogenation for CH_4 generation, the generation of *COH intermediate on Cu(111) surface was also reported in some literatures[7, 8]. To confirm the intermediate in our work, the total energies of the two intermediates on Cu(110) surface are calculated with and without EDTMPA molecule (Supplementary Table 2). Clearly, the calculated total energy of *CHO intermediate is significantly lower than that of *COH intermediate, especially in the presence of EDTMPA molecules (the *CHO intermediate can be more stabilized due to hydrogen bondi, as shown in Supplementary Figs. 16, 17), confirming that the rate-determining step for CH_4 production should be *CO + *H → *CHO in our work.

Supplementary Table 3. The calculated adsorption energy of CO molecule (ΔE(*CO)) and energy change for its hydrogenation (ΔE(*CO→*CHO)) on Cu(110) surface without and with EDTMPA molecule via PBE and revised PBE (RPBE)[9] functional, respectively. The unit is eV.

pure (110) surface	ΔE(*CO)	ΔE(*CO→*CHO)
PBE functional	-1.10	0.44
RPBE functional	-1.01	0.44

EDTMPA	ΔE(*CO)	ΔE(*CO→*CHO)
PBE functional	-1.19	0.10
RPBE functional	-1.04	0.11

The PBE functional is widely used by the theoretical community to explore the CO_2 reduction on Cu-based electrocatalysts, showing well-established reliability[10-12]. However, the RPBE functional proposed by Nørskov and co-workers can provide a more accurate description of the adsorption of small molecules, which is closer to the experimental value, effectively avoiding the over-adsorption problem[9]. Here, we compared their calculated results of the CO adsorption and hydrogenation on Cu(110) surface in Supplementary Table 3. It clearly presents that different functionals have weak effects on CO adsorption and hydrogenation, proving the rationality and feasibility of our data calculated by the PBE functional.

References

1. Marcandalli, G., Goyal, A., Koper, M. T. M. Electrolyte effects on the Faradaic efficiency of CO_2 reduction to CO on a gold electrode. *ACS Catal.* **11**, 4936-4945 (2021).
2. Steiner, T. The hydrogen bond in the solid state. *Angew. Chem. Int. Ed.* **41**, 48-76 (2002).
3. Peterson, A. A., Nørskov, J. K. Activity descriptors for CO_2 electroreduction to methane on transition-metal catalysts. *J. Phys. Chem. Lett.* **3**, 251-258 (2012).
4. Xie, M. S.*, et al.* Amino acid modified copper electrodes for the enhanced selective electroreduction of carbon dioxide towards hydrocarbons. *Energy Environ. Sci.* **9**, 1687-1695 (2016).
5. Peterson, A. A., Abild-Pedersen, F., Studt, F., Rossmeisl, J., Norskov, J. K. How copper catalyzes the electroreduction of carbon dioxide into hydrocarbon fuels. *Energy Environ. Sci.* **3**, 1311-1315 (2010).
6. Nørskov, J. K.*, et al.* Origin of the overpotential for oxygen reduction at a fuel-cell cathode. *J. Phys. Chem. B* **108**, 17886-17892 (2004).
7. Hussain, J., Jónsson, H., Skúlason, E. Calculations of product selectivity in electrochemical CO_2 reduction. *ACS Catal.* **8**, 5240-5249 (2018).
8. Zhao, Q., Martirez, J. M. P., Carter, E. A. Revisiting understanding of electrochemical CO_2 reduction on Cu(111): competing proton-coupled electron transfer reaction mechanisms revealed by embedded correlated wavefunction theory. *J. Am. Chem. Soc.* **143**, 6152-6164 (2021).
9. Hammer, B., Hansen, L. B., Nørskov, J. K. Improved adsorption energetics within density-functional theory using revised Perdew-Burke-Ernzerhof functionals. *Phys. Rev. B* **59**, 7413-7421 (1999).
10. Xiao, H., Cheng, T., Goddard, W. A., Sundararaman, R. Mechanistic explanation of the pH dependence and onset potentials for hydrocarbon products from electrochemical reduction of CO on Cu (111). *J. Am. Chem. Soc.* **138**, 483-486 (2016).
11. Xiao, H., Cheng, T., Goddard, W. A. Atomistic mechanisms underlying selectivities in C_1 and C_2 products from electrochemical reduction of CO on Cu(111). *J. Am. Chem. Soc.* **139**, 130-136 (2017).
12. Nie, X., Esopi, M. R., Janik, M. J., Asthagiri, A. Selectivity of CO_2 reduction on copper electrodes: the role of the kinetics of elementary steps. *Angew. Chem. Int. Ed.* **125**, 2519-2522 (2013).
